# PbS Colloidal Quantum Dots Infrared Solar Cells: Defect Information and Passivation Strategies

**DOI:** 10.1002/smsc.202300062

**Published:** 2023-09-20

**Authors:** Gomaa Mohamed Gomaa Khalaf, Mingyu Li, Jun Yan, Xinzhao Zhao, Tianjun Ma, Hsien-Yi Hsu, Haisheng Song

**Affiliations:** ^1^ Wuhan National Laboratory for Optoelectronics (WNLO) and School of Optical and Electronic Information Huazhong University of Science and Technology (HUST) 1037 Luoyu Road Wuhan Hubei 430074 P. R. China; ^2^ Solar and Space Department National Research Institute of Astronomy and Geophysics (NRIAG) Helwan Cairo 11421 Egypt; ^3^ School of Energy and Environment & Department of Materials Science and Engineering City University of Hong Kong Kowloon Tong Hong Kong 999077 P. R. China; ^4^ Optics Valley Laboratory 1037 Luoyu Road Wuhan Hubei 430074 P. R. China; ^5^ Wenzhou Advanced Manufacturing Technology Research Institute of Huazhong University of Science and Technology Wenzhou Zhejiang 325035 P. R. China

**Keywords:** defects, infrared spectra, passivation strategy, quantum dots (QDs), solar cells

## Abstract

Large‐sized lead sulfide quantum dots (PbS QDs) offer strong absorption in the infrared, making them suitable for bottom cells in tandem devices. However, current QD‐based tandem devices underperform compared to single junction devices. This review focuses on defect information and passivation strategies in large‐sized QD solar cells. Defects from oxidation, polydispersity, and nonbonding sites on the (001) facet during QD synthesis are examined. Ligand‐exchange‐related defects such as tangled atoms, incomplete passivation, and excess ligands are analyzed. Surface and interface defects resulting from solar cell fabrication are also discussed. Strategies including cation exchange, thermodynamic growth, kinetic growth, and mixed halide ligands are summarized. Post‐treatment approaches could also help to address surface and interface defects. Large‐sized PbS‐QDs show promise as infrared radiation absorbers. Overcoming defects and implementing effective passivation strategies are crucial for single junction and tandem solar cells.

## Graphical abstract

Large‐sized lead sulfide quantum dots (PbS QDs) can harvest and convert the power of infrared (IR) light. These features along with solution processability advocate it as a bottom subcell in tandem devices. However, large‐sized QDs are more prone to varied defects. Thus, PbS QDs defect information and passivation strategies are significant issues for introducing substantial IR solar cells.

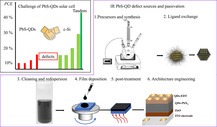

## 1 Introduction

**Figure 1:**
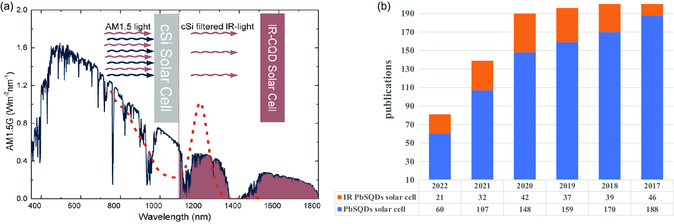
a) Comparison between the absorbed radiation by CQDs and the AM1.5G spectrum showing the well‐matching of IR CQDs solar cells for tandem devices with c‐Si or perovskite. Reproduced with permission.^[^ ^103^ ^]^ Copyright 2019, Wiley‐VCH. b) Bar chart for the number of publications that appeared in the Web of Science with keywords PbS quantum dots and (solar cells/infrared solar cells).

Photovoltaic (PV) devices that convert sunlight to electricity become essential to cope the related crises. Crystalline silicon (c‐Si) solar cells are currently dominant in the photovoltaic (PV) commercial market. However, their power conversion efficiency (PCE) is limited by the Shockley–Queisser's (SQ) efficiency constraint. These solar cells only utilize approximately 15% of the solar spectrum.^[^ ^1^ ^]^ Single junction solar cells have met their limitation, and the next generation PV technology is tandem structure to surpass the boundary 33% PCE.^[^ ^2^, ^3^ ^]^ Tandem solar cells of three junctions have reached about 39% PCE,^[^ ^4^ ^]^ by expanding the spectral response to infrared radiation (IR), half of the total incident solar radiation (**Figure** **1**a).^[^ ^5^ ^]^ Low‐cost tandem solar cells with broad spectral response are encouraged to fill the global energy deficit, but tandem construction at a low cost is still a competitive challenge. So far, high‐efficiency devices are fabricated through expensive techniques such as epitaxy.^[^ ^6^, ^7^ ^]^

Among the next‐generation solar cells, PbS colloidal quantum dots (PbS‐QDs) have surfaced due to their outstanding characteristics for cost‐effective and efficient PV candidate.^[^ ^8^, ^9^, ^10^, ^11^ ^]^ These features include IR absorption,^[^ ^12^, ^13^, ^14^, ^15^, ^16^, ^17^ ^]^ bandgap tunability,^[^ ^18^, ^19^ ^]^ multiexciton generation,^[^ ^20^, ^21^, ^22^, ^23^, ^24^ ^]^ solution processability,^[^ ^10^, ^25^, ^26^, ^27^ ^]^ the controllable size,^[^ ^19^, ^28^, ^29^ ^]^ and quantum confinement.^[^ ^5^, ^30^ ^]^ The large QDs (LQDs) booked the bottom subcell in tandem due to their broad IR absorption spectra.^[^ ^31^, ^32^, ^33^, ^34^, ^35^ ^]^ Thus, the low‐cost tandem devices could be realized by combining perovskite or c‐Si with PbS‐QDs, demonstrating high potential in PCE and cost.^[^ ^30^, ^34^ ^]^ Unfortunately, the PbS‐QDs single junction solar cell has PCE still lower than the conventional Si‐based PV cells.^[^ ^11^, ^36^ ^]^ Furthermore, the tandem structure based on PbS‐QDs achieves lower efficiency than the single junction. Because the short minority carrier lifetime and short diffusion length of QD absorber limit their device performance.^[^ ^32^, ^37^, ^38^, ^39^, ^40^, ^41^, ^42^ ^]^ That means PbS‐QDs could contribute more for tandem construction after the defects’ density reduction and the charges diffusion length extension.

Studying PbS‐QDs defects along with the efficient passivation strategies is the bottleneck to seize the solar energy loss.^[^ ^43^, ^44^ ^]^ To illustrate the influence of defects on PbS‐QDs, Figure 1b shows the annual number of reported studies about PbS‐QDs solar cells during the last 5 years. It reveals a continued interest in PbS‐QDs solar cell research over time. However, the decrease in publications indicates that this field needs a breakthrough in the passivation of PbS‐QDs. From the charge diffusion length standpoint, PbS‐QDs have the maximum absorption at a thickness close to 300 nm, and the thicker absorber causes degradation in the PV parameters.^[^ ^32^, ^45^ ^]^ The participation in tandem structure demands an absorber permitting long carrier diffusion length, which also requires extended carrier lifetime and high mobility. The PbS‐QDs defects promote recombination centers and mid‐gap states, which degrade the factors mentioned earlier.^[^ ^8^, ^46^ ^]^ The route to increase the PbS‐QDs efficiency in the IR region is the surface and interface passivation.^[^ ^14^, ^47^ ^]^ The defects raise a barrier for PbS‐QDs to participate in the tandem structures, so the apparatus to remove this barrier is the investigation of passivation strategies.

Here, the factors that withstand the PbS‐QDs solar cells’ efficiency are spotlighted. In particular, this review is formed of four main sections. First, it overviews the PbS‐QDs, the unique property of size‐dependent bandgap, and their application in IR absorption. After that, the authors describe the PbS‐QDs defects either in the solution synthesis or the device fabrication process. Then, the approaches for passivation and recombination suppression are summarized in detail. Ultimately, the challenges for industrial production and the future of PbS‐QDs solar cells are highlighted.

### 1.1 Engineering the PbS‐QDs Size for IR Absorption

**Figure 2:**
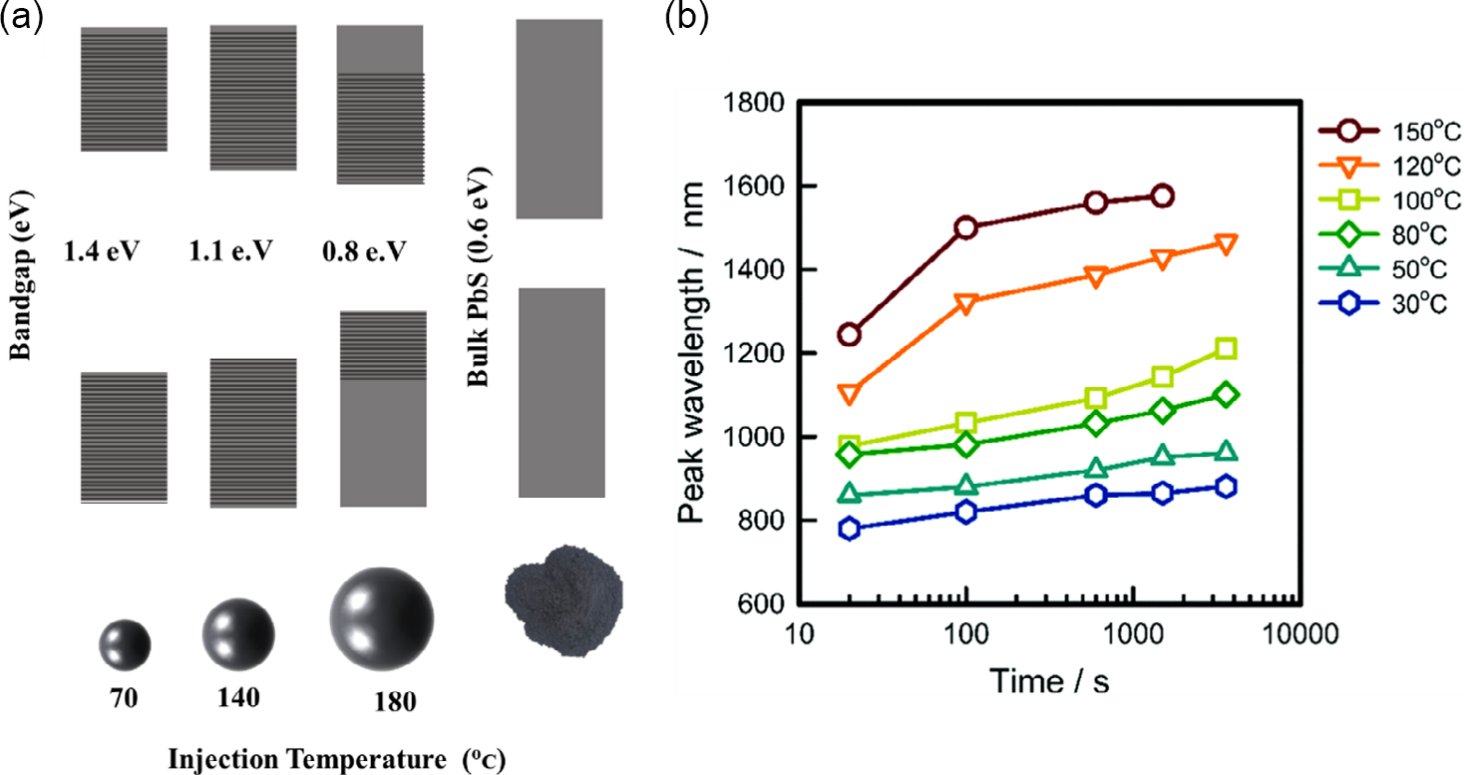
a) Schematic diagram depicting the controllable bandgap due to tuneable QDs size and synthesis temperature. The data are extracted from the previous studies.^[^ ^49^, ^126^ ^]^ b) The change of the first exciton peak wavelength of PbS‐QDs prepared at different temperatures. b) Reproduced with permission.^[^ ^28^ ^]^ Copyright 2017, Royal Society of Chemistry.

PbS‐QDs are assembled in a PbS core and an external shell of ligand molecules.^[^ ^48^ ^]^ These create a bridge between the QDs, which plays a significant role in QDs properties.^[^ ^9^, ^44^ ^]^ Regarding the bandgap tunability, controlling the dot diameter yields an adjustable bandgap^[^ ^49^, ^50^ ^]^ according to Moreels et al. illustrated in **Figure** **2**a.^[^ ^29^ ^]^ The relation between the QDs diameter (*d*) and the corresponding bandgap (*E*_g_) is depicted in Equation (1).^[^ ^51^, ^52^ ^]^ Therefore, the bandgap required to harvest IR light is associated with QDs diameter between 4.2 and 7.4 nm.^[^ ^28^, ^53^ ^]^ Commonly, the QDs diameter is tunable by adjusting the injection temperature of the hot injection method in the QDs synthesis process.^[^ ^28^ ^]^ The first exciton peaks for PbS‐QDs synthesized at different temperature are depicted in Figure 2b, which reveals that the long wavelength is attributed to the higher synthesis temperature of up to 150 ºC. This evidences the relation between synthesis temperature, QDs size, and bandgap.^[^ ^28^ ^]^ The PbS‐QDs optoelectronic properties are tunned by controlling the physicochemical properties, which are restrained by the synthesizing raw materials, ligands, and the reaction conditions.

(1) $$E_{g}=0.41+\frac{1}{0.0252d^{2}+0.283d}$$

## 2 Defects in PbS‐QDs Solar Cells

### 2.1 The Effects of Defects

**Figure 3:**
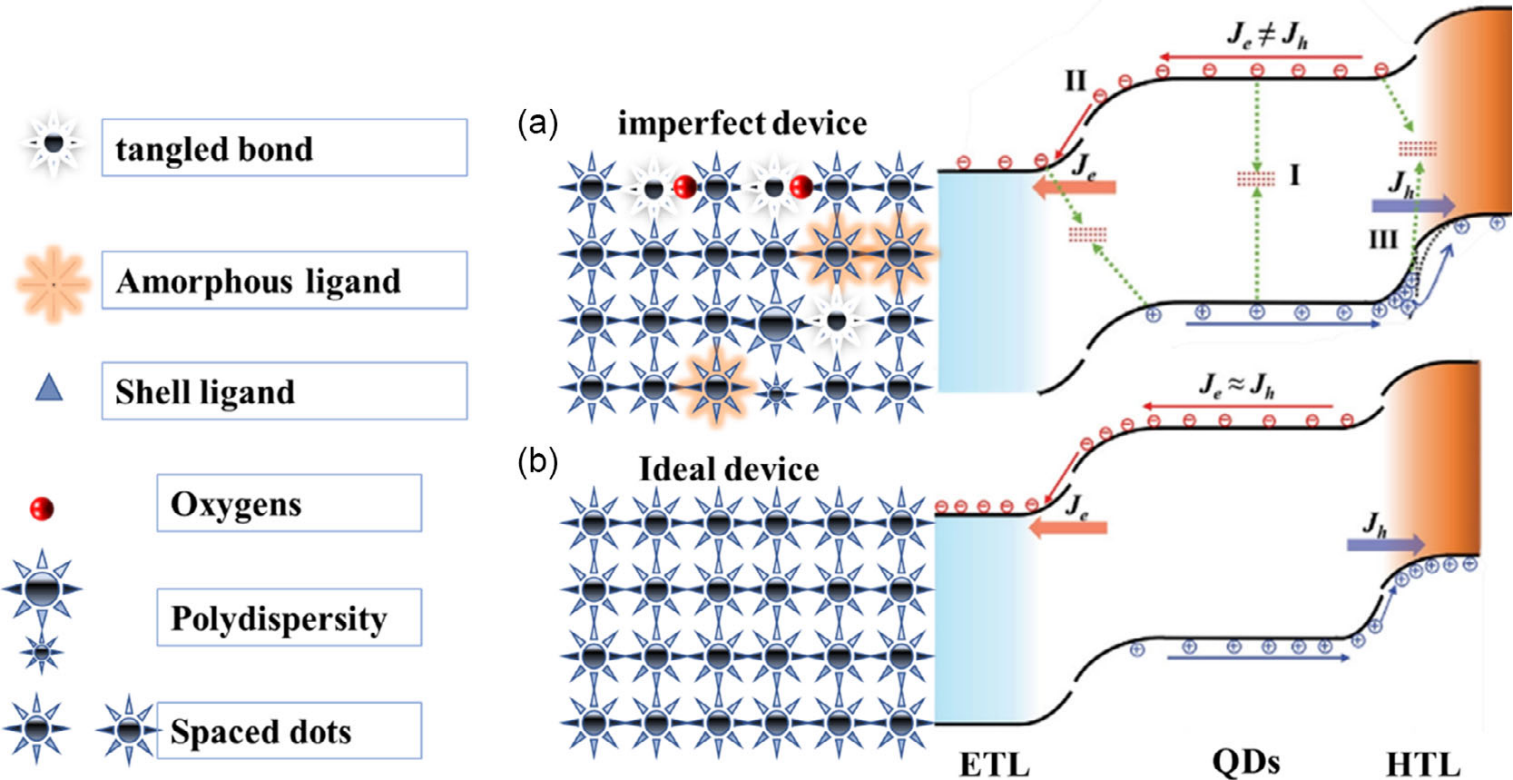
a) Schematic portrait of the different types of defects in the PbS‐QDs films and device. b) Band bending diagram for the ideal PbS‐QDs device depicts the perfect absorber film. a,b) Adapted with permission.^[^ ^8^ ^]^ Copyright 2022, Wiley‐VCH.

The influence of defects on the solar cell's performance can be briefed in the low photo‐electric current, short carrier lifetime, and deficit in the open circuit voltage. Traps induce inequity of charge carriers at both electrodes and stimulate irradiative recombination.^[^ ^8^ ^]^ Thus, the unbalance between electron and hole mobilities in the absorber increases *J*_sc_ and diminishes the FF forming hysteresis in the device's *J–V* curve. **Figure** **3**a,b shows the schematic comparison between the imperfect device in Figure 3a with the perfect solar cell with fully passivated defects, as shown in Figure 3b. The *J–V* hysteresis directly results from nonhomogeneous carrier distribution and accommodation of charges at its opposite polarity. For effect on the *V*_oc_, the ligand deficiency in the outer shell of QDs notably devolves the carrier's transfer. Then it causes a significant deficit in *V*_oc_, which is reported as an essential drawback of PbS‐QDs solar cells.^[^ ^54^, ^55^ ^]^ However, the existence of long organic chains during the growth of PbS‐QDs is not considered as a defect in the device. It is one of the prevalent generators for mid‐gap states PbS‐QDs.^[^ ^56^, ^57^ ^]^

From the mobility standpoint, the remaining indigenous or introduced ligands at the QDs surface hinder carrier mobility. This causes the short diffusion length and short lifetime.^[^ ^58^ ^]^ Also, the PCE directly increases with the hole mobility increasing from 10^−4^ to 1 cm^2^ V^−1^ s^−1^. It increases with the electron mobility from 6.3 × 10^−4^ to 4 × 10^−3^ cm^2^ V^−1^ s^−1^ and any further enhancement in electron mobility decreases the performance.^[^ ^59^ ^]^ This critical value is often referred to as the “mobility cutoff”. The mobility cutoff is related to the balance between charge extraction and recombination in the device. When the electron mobility is below the mobility cutoff, charge extraction is limited, resulting in a decrease in the overall current density and PCE of the solar cell. In contrast, when the electron mobility exceeds the mobility cutoff, charge extraction becomes more efficient, but other factors like recombination can start to dominate and lead to a decrease in PCE.

The effect of traps density on the PCE reduction is higher at the QDs/hole extraction layer (HTL) interface than in the other two locations (i.e., absorber layer and electron transport layer (ETL)/QDs interface).^[^ ^8^ ^]^ In particular, the optimum efficiency of 15% is achievable by decreasing the defects at QDs/HTL to 1.5 × 10^13^ cm^−3^.^[^ ^8^ ^]^ While the influence of defects on the performance is neglected at the values of 2.1 × 10^15^ and 1 × 10^13^ cm^−3^ in the absorber and the ETL/QDs interface, respectively.^[^ ^8^ ^]^

### 2.2 The Sources of Defects

The defects of PbS‐QDs solar cells are induced during the two principles stages, the QDs synthesis and the fabrication of the device. In the first stage, defects are created by incomplete passivation, such as uncoordinated lead ions. Thus, vacancies in the outer shell of the QDs are generated in part (I), as shown in Figure 3a.^[^ ^60^ ^]^ The next type is also attributed to the QDs synthesis, which is described as the nonhomogeneous manner for the sulfur anion inducing the imbalance between sulfur and lead. Likewise, the absence of homogeneity and stoichiometry also degrades stability and efficiency.^[^ ^61^ ^]^ Finally, the defects at PbS‐QDs are introduced during ligand exchange process. To illustrate, the layer‐by‐layer ligand exchange causes surface oxidation. Like in the solution ligand, the excess of ligands (e.g., PbI_2_) is converted into an amorphous phase at the boundaries or the space between dots.^[^ ^62^ ^]^

The second stage is the device fabrication, where the defects are contained in the two positions called ETL/PbS‐QDs interfaces and the PbS‐QDs /HTL interface^[^ ^8^ ^]^ The interface defects between QDs and the ETL or HTL attracts the attention of many research groups.^[^ ^9^, ^63^, ^64^ ^]^ These vacancies on the PbS surface and the interface with ETL are generated by interacting with the ambient atmosphere and the influence of the relative humidity.^[^ ^5^, ^20^ ^]^ There are cracks and disorders in the ZnO surface because the strong solvents in the QDs solution interact with the ZnO surface during the absorber deposition.^[^ ^8^ ^]^ The defects on the QDs’ surface are generated by the Auger process and commonly cause a deficit in the device voltage.

### 2.3 Defects Detection

The characterization of traps is necessary step for their passivation,^[^ ^65^ ^]^ so the impedance spectroscopy (IS) is widely applied for this purpose. The IS system comprises an illumination source and frequency analysis of alternative current (AC) behavior under working conditions. The IS role is to monitor the dependent parameter, such as electric current *J*(*ω*) due to the applied voltage *V*(*ω*), where all the parameters are angular frequency dependent.^[^ ^66^, ^67^ ^]^ Analyzing the measured data gives insights into the dynamic of charges inside the layers and at the interfaces, the depletion region attributed to the density of the traps, the carrier lifetime, and the film's conductivity under dark and illumination conditions. Accordingly, it evaluates the equivalent circuit for solar cells.^[^ ^68^, ^69^ ^]^ Overall, the IS is a convenient technique to characterize the defects in the PbS‐QDs solar cells by monitoring the device response to the AC instead of direct current (DC) parameters.

Regarding the application of IS in detecting defects, figuring the actual impedance of the solar cell on the x‐axis and the imaginary impedance on the y‐axis deduce the semicircle spectrum known as the Nyquist plot. The radius of this plot is proportional to the carrier recombination resistance.^[^ ^68^ ^]^ In the PbS‐QDs, this method explained the distribution of charge in the device and the mechanism for its recombination.^[^ ^27^ ^]^Also, it reveals that the trap's density is around 3.2 × 10^16^ cm^−3^ eV^−1^ at about 0.34 eV under the conduction band.^[^ ^65^ ^]^ As expressed, the IS is a promising method to measure the defect by analyzing the data in various figures giving information about the carrier distribution, transformation, and recombination.

Furthermore, photoluminescence (PL) spectroscopy is used to detect the trap states of PbS‐QDs as it is a sensitive and nondestructive method for optoelectronic properties investigation. These are the radiations emitted from a molecule in its excited state. The monochromatic light has greater energy than the semiconductor *E*_g_ absorbed, generating excitons. When these excitons recombine, photons are emitted having energy equal to the *E*_g_ of the material. Such a type of recombination is also called radiative recombination. When the defects trap charges, the emitted photon's energy is less than the bandgap energy.

## 3 Passivation Strategies from Synthesis and Device Fabrication

As mentioned, all the steps undergo different types of defects. Accordingly, each step is receiving a suitable passivation. The first PbS‐QDs device was realized by Plass et al. two decades ago, getting a PCE of about 0.5%.^[^ ^70^ ^]^ After that, many articles with significant performance that mainly acquired by several conditions,^[^ ^71^ ^]^ such as the QDs synthesis including the ligand types,^[^ ^20^, ^44^, ^68^, ^72^, ^73^ ^]^ the device architecture, films deposition,^[^ ^50^, ^74^ ^]^ passivation degree of surfaces,^[^ ^64^, ^75^, ^76^ ^]^ and quantum dot quality.^[^ ^11^, ^22^ ^]^ This section presents the progress in synthesis methods for LQDs free of trap states.^[^ ^48^, ^77^, ^78^ ^]^ Additionally, the used precursors affect QDs quality, and device performance, particularly the use of lead oxide as a lead source in the QDs synthesis is more defective than lead acetate.^[^ ^79^ ^]^ Also, the outstanding ligands for LQDs rather than conventional ones are touched. For example, the phase transfer exchange was concluded as a promising ligand exchange for obtaining high‐passivated and conductive QDs.^[^ ^11^, ^80^ ^]^ Then, the device architecture is sentenced to modifications so as to avoid the trap states. Finally, the passivation strategies serve all the detailed steps from the QDs synthesis and the device fabrication (i.e., the precursors, QDs preparation, ligands, the structure surfaces, and interfaces), which are described in the following subsections.

### 3.1 Passivations Based on QDs Synthesis

The large QDs solar cells with a narrow size distribution, monodispersity, and high pure surface facets are encouraged for PV fabrication. Hence, pioneering methods for QDs synthesis with the preceding properties are cutting‐edge attempts. The hot injection under controlled temperature and nitrogen gas is the conventional method for QDs synthesis. However, the application for LQDs has a broader size distribution. The surface defects are a massive presence, as well as the exposure of (001) facets. In the produced QDs,^[^ ^53^, ^81^, ^82^ ^]^ traditional method of preparing QDs localizes the known defects in the LQDs, so here is a synopsis of the researchers’ efforts to provide alternative methods to overcome this issue.

#### 3.1.1 The Cation Exchange Method

**Figure 4:**
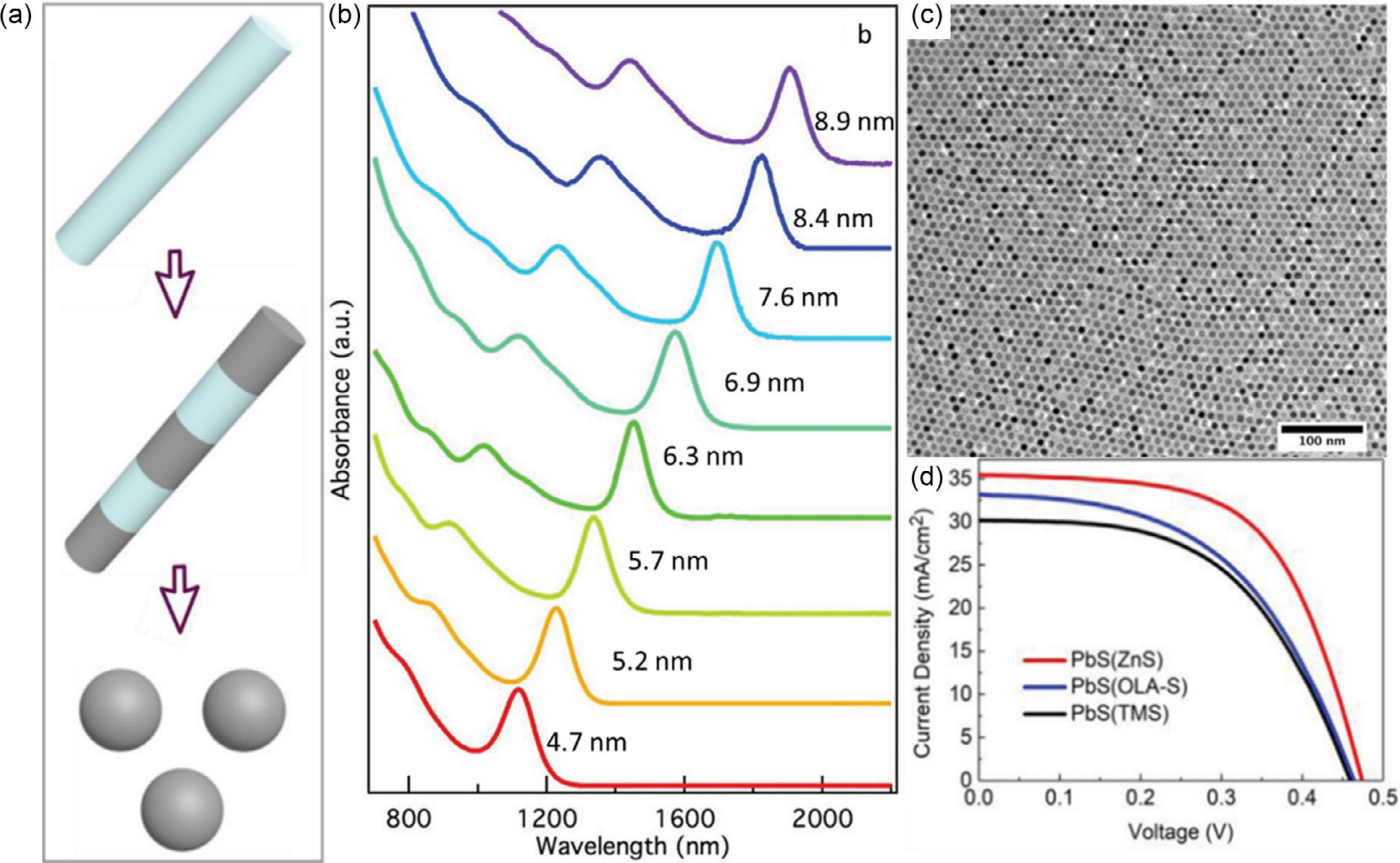
The cation exchange for PbS‐QDs synthesis. a) Mutation mechanism from ZnS NRs to PbS‐QDs diagram displays the modification process. b) First‐exciton peaks for the different size QDs. c) TEM image for the QDs. d) *J*–*V* characterization of the 1.4 eV bandgap device under AM 1.5, compared to the devices based on PbS (TMS) and PbS (OLA‐S). a–d) Reproduced with permission.^[^ ^53^ ^]^ Copyright 2020, John Wiley and Sons.

The cation exchange has recently emerged in the investigation for nanomaterials synthesis for various applications.^[^ ^83^ ^]^ For LQDs synthesis, this method was modified by Zhang et al. using ZnS nanorods, as shown in **Figure** **4**a.^[^ ^53^ ^]^ The QDs size is controlled by optimizing the reaction temperature and synthesis time, as shown in Figure 4b. By increasing the reaction time, the PbS dots arise larger, and the NRs cannot maintain the interfacial strain. Hence, they break into smaller rods and then finally into single PbS‐QDs. The PbS‐QDs prepared in this style give high stability under ambient conditions. The polydispersity, known as a trap source, has been wholly processed, as shown in Figure 4c. utilizing this efficient surface passivation, these PbS‐QDs devices obtain high performance with a PCE of 10.0% under AM 1.5 solar illumination. The *J–V* curves shown in Figure 4d remarkably reveal the superiority of cation exchange to its counterparts, such as using bis(trimethylsilyl methyl) sulfide (TMS) or using sulfur powder in oleylamine (OLA‐S).^[^ ^53^ ^]^ In short, the cation exchange overcomes the usual disadvantages in the conventional methods of preparing PbS LQDs.

**Figure 5:**
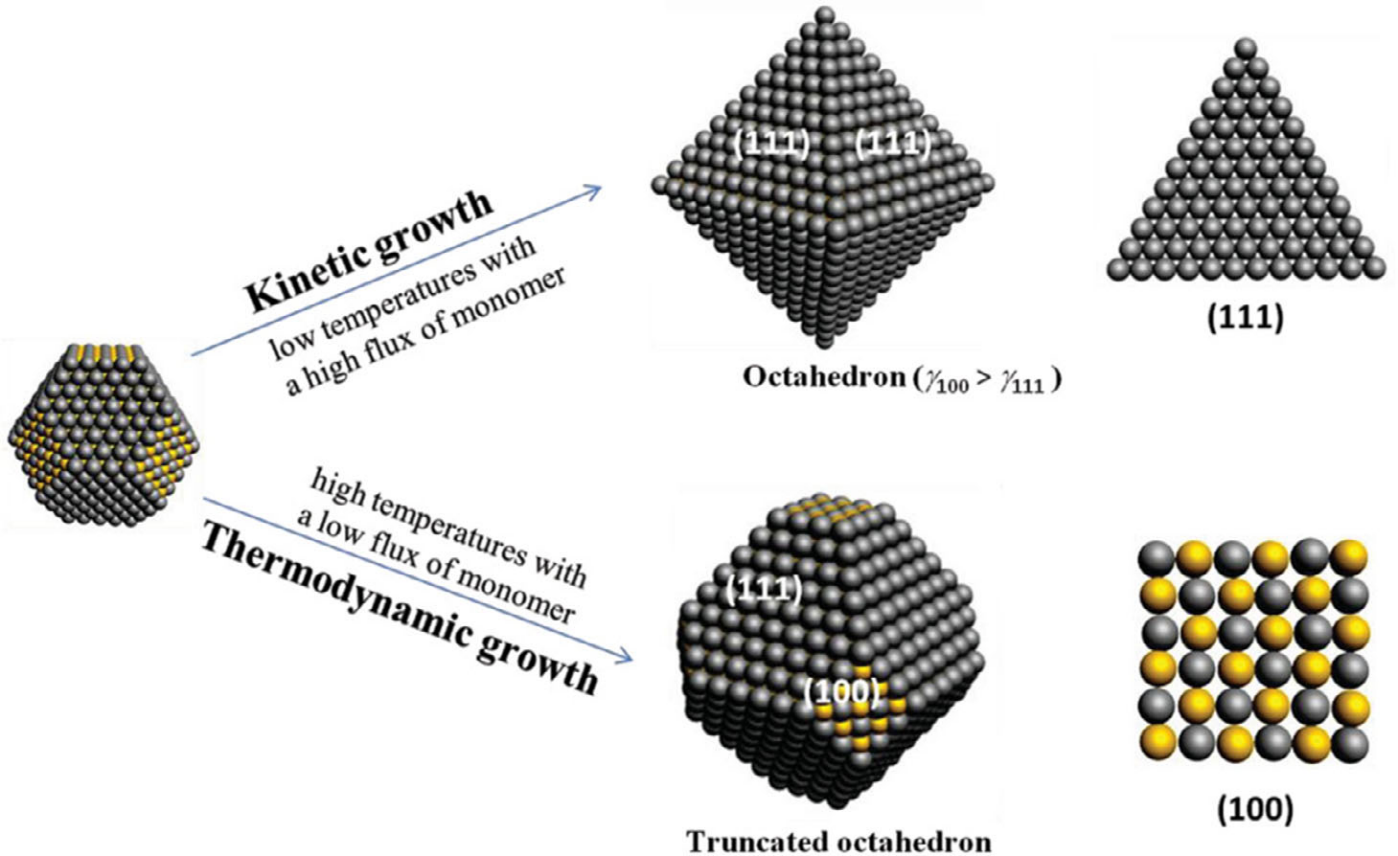
Schematic illustration of two approaches to control the terminating facets of PbS‐QDs. The black and yellow spheres symbolize the Pb and S atoms, respectively. Reproduced with permission.^[^ ^84^ ^]^ Copyright 2020, Wiley‐VCH.

How to control facet; increasing the PbS (100) to the Pb (111) facets ratio in LQDs causes trap state formation due to irreversible surface oxidation.^[^ ^15^, ^84^, ^85^ ^]^ The complete passivation strategies remedy surface oxidation. For controlling the facets in cation exchange QDs synthesis, the ZnS QDs are used as seeds, as aforementioned. They are controlling the QDs synthesis, kinetics, and thermodynamics growth, as shown in **Figure** **5**.^[^ ^86^ ^]^ In particular, the synthesis under low temperatures and high oversaturation of monomers is known as kinetic growth, and the resultants are almost octahedral nanoparticles composed of (111) facets. For reduced octahedral nanoparticles, thermodynamic growth is preferred where the synthesis temperature is comparatively high and low concentration of monomers. Hence, a spherical shape raises octahedral nanoparticles with dense (100) facets. Compared to PbS‐QDs from thermodynamics‐dominated growth, the kinetic growth produces PbS‐QDs with fewer (100) facets, demonstrating fewer trap states and contributing to more beneficial PV performance with a PCE of 11.5%.^[^ ^83^ ^]^ Notably, this facet‐controlling strategy is held for the small‐size QDs ≤ 3 nm diameter, where the ZnS QDs are used as the reaction seed. For the large‐size PbS‐QDs, the ZnS nanorod is used as the seed for the reaction.

#### 3.1.2 Direct Synthesis of QDs

**Figure 6:**
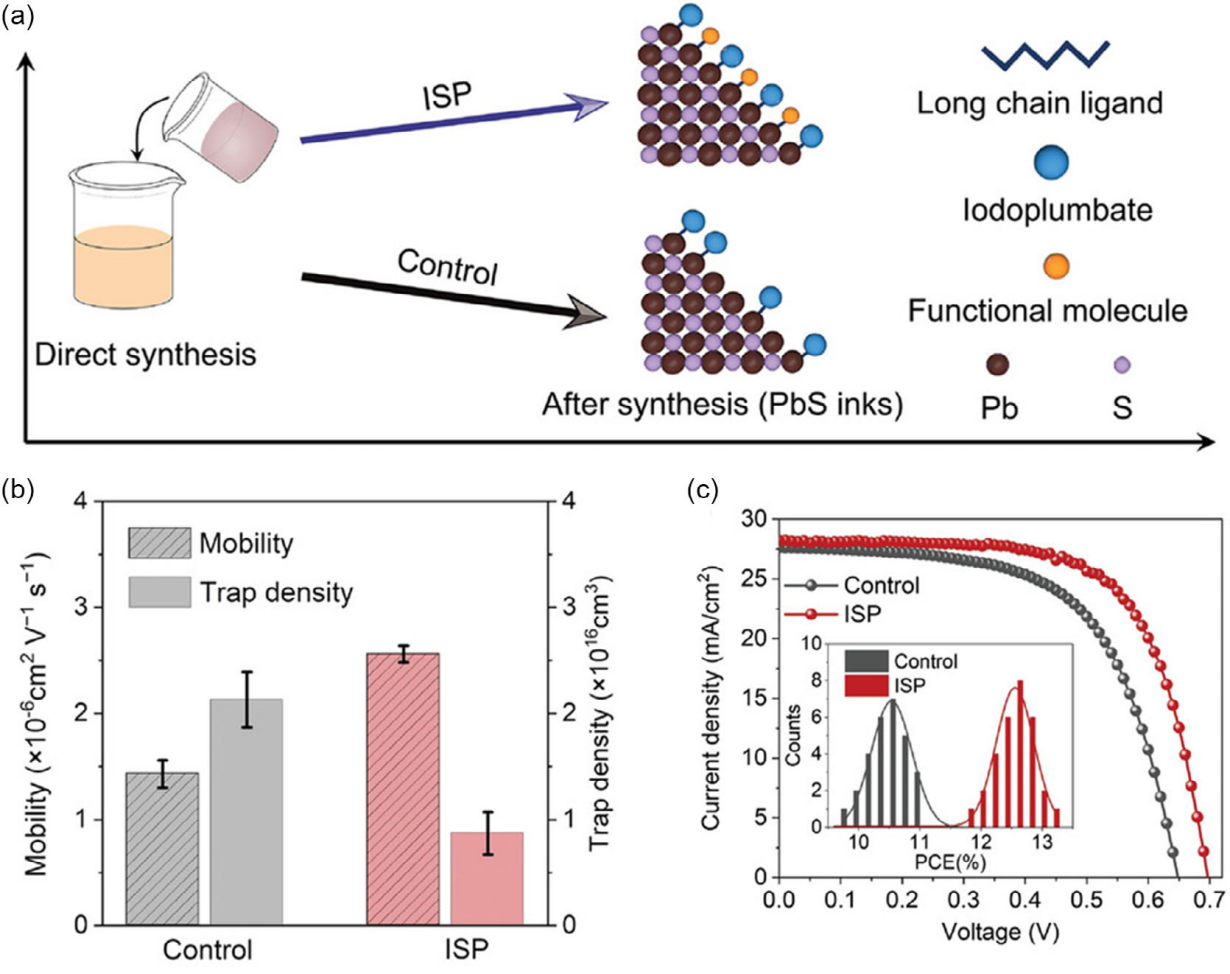
The merged passivation for both QDs synthesis and ligand exchange. a) Schematic illustration for the direct synthesis method for QDs along with the ISP modification. b) Trap density and mobility for devices based on QDs synthesized. c) Comparing the *J*–*V* curves for DI and ISP methods. a–c) Reproduced with permission.^[^ ^88^ ^]^ Copyright 2022, Wiley‐VCH.

QDs based on merging the QDs synthesis with ligand exchange have recently introduced.^[^ ^87^, ^88^ ^]^ Succinctly, PbI_2_ dissolved in a mixture of DMF and diphenyl thiourea (DphTA). Then, butylamine (BA) is expeditiously injected; afterward, the solution color turns black. This method is defined as direct synthesis and is schematically shown in **Figure** **6**a compared with the in situ synergistic passivation (ISP). Bifunctional ligand molecules are added during synthesis and before the BA injection in the latter method.^[^ ^88^ ^]^ Moreover, in the shortened synthesis, its ink achieved a competitive carrier photoactivity, such as decreasing the trap states density from 2.13 ± 0.26 × 10^16^ cm^−3^ for the control sample to 8.70 ± 0.20 × 10^15^ cm^−3^ for the ISP approach. This enhancement in PbS‐QDs traps and charge mobility is shown in Figure 6b. Trap reduction improves PV parameters and efficiency, as shown in Figure 6c. Interestingly, the open circuit voltage deficit is reduced to 0.35 eV, the highest benefit of *V*_oc_ reported till now for efficient (more than 10% PCE) PbS‐QDs solar cells.^[^ ^88^ ^]^ It is notable that the direct method is crucial for defects suppresion because in this method the ligand exchange step is skipped. During this step there is a change for QDs surface oxidation. In contrast, the accurate amount of ligand required for a certain amount of QDs solution is diffcult to confirm. Including the ligand exchange process within the QDs synthesis is a great step toward the QDs solar cell free of defects.

The QDs solution receives further treatment after synthesis for reconstructing the atoms and reducing the defects at the QDs surface. In particular, treating the ligand's trouble by solution annealing removes the remaining dislocation after organic ligand desorption and keeps the QDs more stable.^[^ ^89^ ^]^ Hence, the carrier's mobility for the QDs underwent solution annealing is doubled from 2.40 × 10^−3^ to 1.09 × 10^−3^ cm^2^ V^−1^s^−1^ for the control and annealed one, respectively.^[^ ^89^ ^]^ So, the synthesis methods play an essential role in the PbS‐QDs defects, either prevention or passivation. Conveniently, the trap states are limited, and the optimum facets are achievable.

### 3.2 Passivation Strategies Based on Ligand Exchange

Ligand exchange is the origin of several defects, such as dimer QDs. The change of the ligands causes a change in the trapped carrier density from 0.1 to 10^−4^ per QDs.^[^ ^90^, ^91^ ^]^ The defects in the QDs solar cells are almost attributed to the surface, so most trap states fall behind the ligand exchange process. In addition, the reactivity of ligands with PbS‐QDs is size dependent, so the large PbS‐QDs require unique ligand exchange.^[^ ^92^ ^]^ Finding functionalized ligands without steric hindrance has been considered over the years.^[^ ^55^, ^93^, ^94^ ^]^ Briefly, the convenient ligands for PbS‐QDs include atomic halides, lead halides, ionic sulfur, and perovskite, etc. **Table** **1** is a comprehensive summary of the ligands used for PbS‐QDs shells over the course of the recent 5 years. This section highlights the convenient ligands for LQDs and their influence on improving the device's performance. Furthermore, the ligand's solvent has received some efforts to select the optimum property of the long (OA) removal without causing halogen vacancies. The optimum solvent mediated is significant in improving the PV parameters of the QDs solar cells. This enhancement was caused by reduction in the defects density from 0.64 × 10^15^ cm^−3^ with the use of EtOAc/CI‐based QDs to 0.5 × 10^15^ cm^−3^.^[^ ^95^ ^]^

**Table 1:** The ligands capped the PbS‐QDs along with their influence in all the physical parameters, such as the PV parameters and carriers’ mobility, lifetime, etc

Ligand	*V*_OC_ [V]	*J*_sc_ [mA cm^−2^]	FF [%]	PCE [%]	*E*_g_ [eV]	*T* [ns]	*μ* [cm^2 ^v^−1^s^−1^]	Trap‐density [cm^−3^ eV^−1^]	Size [nm]	IND [nm]	References
MPA	0.7	28.22	67.35	13.30		8.83	2.56 10^−6^	8.7 × 10^15^			[88]/2022
FAPb (IBr)_3_	0.64	31.1	65.9	13.10			9.2 × 10^−3^	8.1 × 10^18^			[8]/2022
PbI_2_:PbBr_2 _+ *X*‐PEAI	0.62	34.48	66.80	11.80	1.30	1.8	1.4 × 10^−3^		3.00	0.80	[62]/2022
PbI_2_:PbBr_2_	0.49	34.48	61.80	10.47	0.70	18		4.2 × 10^16^	4.50		[12]/2022
PbI_2_:PbBr_2 _+ *X*‐PEAI	0.62	25.08	70.00	11.00	1.36		2.4 × 10^−3^	8.3 × 10^15^	2.96		[89]/2022
PbI_2_ + 10 mm CPTa)	0.63	31.40	53.65	10.60					3.19		[102]/2021
MAPbI_3_	0.57	25.00	0.61	8.70	1.39				2.70		[117]/2021
PbI_2_ + KI_3_	0.64	27.7	68.00	12.10		1.50		1.03 × 10^16^			[61]/2021
PbI_2_ + MAClb)	0.63	30.9	63.9	12.40	1.19	4	2.4 × 10^−2^	1.4 × 10^17^	3.5		[46]/2020
PbI_3_^−^ + 40 mm CPTa)	0.42	33.74	57.01	8.07	1.00	0.90	1.9 × 10^−3^	1.1 × 10^17^	4		[26]/2020
PbI_3_^−^ + MPEa)	0.61	30.30	52.00	9.60			1.2 × 10^−3^	1.96 × 10^16^			[97]/2020
PbI_2_:PbBr_2 _+ FABra)	0.65	30.00	71.00	13.80	1.29	72	3.7 × 10^−1^			3.20	[11]/2020
PbBr	0.64	29.50	66.00	12.50			3 × 10^−2^	1.5 × 10^15^			[93]/2020
tri‐EAHIa)	0.58	24.80	67.00	9.84	1.40		1.2 × 10^−3^	4.90 × 10^16^	2.90		[127]/2019
Pb*X* + formate	0.47	28.3	54.2	7.20	1.05		2 × 10^−2^	8 × 10^15^		3.80	[16]/2019
CTABa) + xanthate	0.43	26.04	44.00	4.96			5 × 10^−1^				[107]/2018
CsPbI_3_	0.64	24.50	67.00	10.50				2.61 × 10^16^			[75]/2018
PbI_2_ + AA	0.1	27.23	67	11.28	1.32				2.9	3.21	[69]/2017
PDMIIa)	0.68	24.11	67.00	10.99			9.7 × 10^−4^	1.20 × 10^17^			[128]/2017
ZnI_2_ + MPAa)	0.65	24.48	61.00	9.92	1.18		3.7 × 10^−3^				[55]/2017
ZnI_2_ + MPAa)	0.69	20.84	53.00	7.62	1.20						[105]/2017

a) CPT: 3‐chloro‐1‐propanethiol; MPE: 3‐methyl mercapto propionate; tri‐EAHI: triethylamine hydroiodide; CTAB: cetyltrimethylammonium bromide; PDMII: 1‐propyl‐2,3‐dimethyl imidazolium iodide; MPA: 3‐mercaptopropionic acid; FAI: formamidine iodide; PEAI: phenylmethyl‐ ammonium iodide; X: always refers to the halide's mixture (I, Br, or Cl); IND: refers to the interdot distance; *E*_g_: PbSQDs bandgap; *T* refers to the carrier lifetime, and *μ* refers to the charge mobility;

b) This further MAX is exchanged by the solid‐state method.

#### 3.2.1 The Inorganic Halides

In addition to being utterly interactive with PbS‐QDs, the inorganic anions shave defects and prevent oxidation by filling the surface vacancies.^[^ ^5^, ^96^, ^97^ ^]^ These surface vacancies are common defect in all the QDs materials. To illustrate, perovskites have been treated by introducing organic materials that provide the adequate iodine ions, for example, tert‐butyl iodide (TBI) and nucleophile trioctylphosphine were introduced during the postpurification of the inorganic CsPbI_3_ QDs to produce surface matrix curing (SMC).^[^ ^98^ ^]^ The ligand trend had partially shifted to inorganic one 9 years ago by introducing atomic passivation with a record efficiency of 5.1%.^[^ ^20^ ^]^ Then several types were introduced, and we are reviewing them in light of the adaptation for LQDs. Because the ligands should be functionalized with the sizes over 4 nm size.^[^ ^53^ ^]^ First, as it holds a high ratio of (100) facets, they are ambiently unusable. Then, progress in the ligand exchange is also unique since the crystal planes of LQDs are different from the small dots. Hence, LQDs passivation is essential for air stability enhancement and aggregation protection during the ligand exchange process.^[^ ^85^, ^99^ ^]^

**Figure 7:**
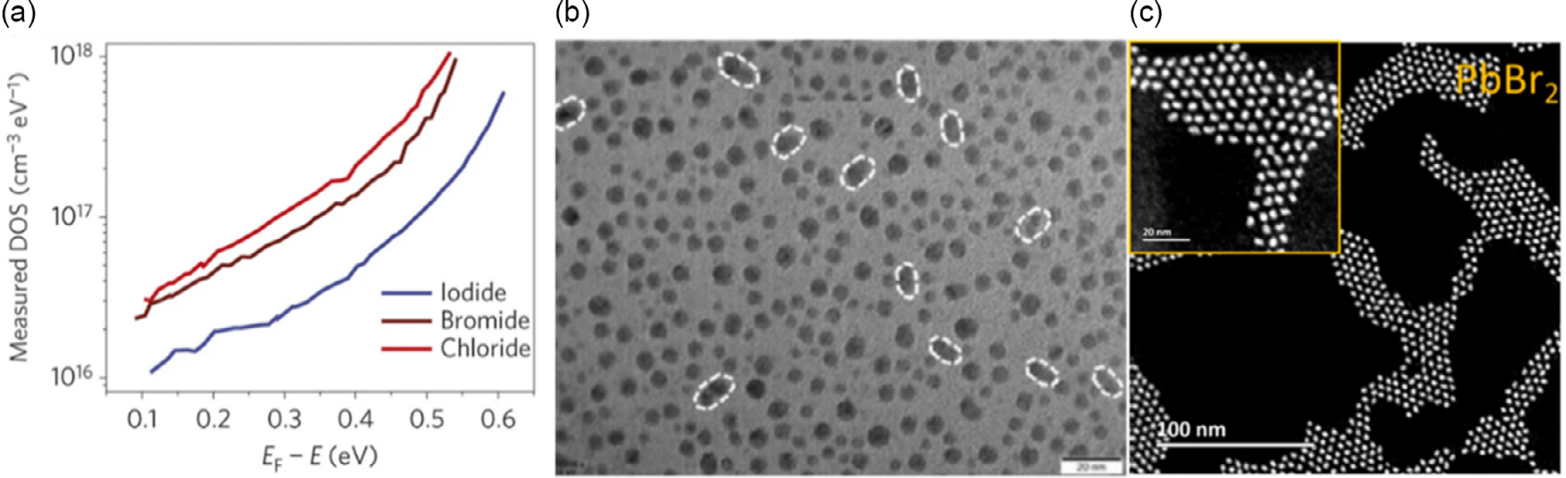
a) Comparison between the ionic halide ligand from a defect density standpoint. Reproduced with permission.^[^ ^100^ ^]^ Copyrights 2014, Springer Nature. b) TEM image for the PbS‐QDs with lead iodide ligand. Reproduced with permission.^[^ ^26^ ^]^ Copyright 2020, American Chemical Society. c) Scanning transmission electron microscopy (STEM) image of PbS‐QDs with PbBr_2_ ligand. Reproduced with permission^[^ ^93^ ^]^ Copyright 2020, American Chemical Society.

First, the halides, mixed or separated, are the most common ligand.^[^ ^20^, ^22^, ^26^, ^100^ ^]^ The hole's mobility in PbS‐QDs is close to 10^−4^ cm^2^ V^−1^ s^−1^ for all the halides ligand.^[^ ^101^ ^]^ Also, the halide ligands decrease the interdot distance and treat the tangled sulfur bond on the PbS‐QDs surface.^[^ ^20^, ^22^ ^]^ The lowest trap states of 1.5 × 10^15^ cm^−3^ eV^−1^ are based on lead halides.^[^ ^93^ ^]^ **Figure** **7**a shows the density of states at the depletion region of PbS‐QDs. It reveals that the iodine ligands cause the lowest trap density compared to chlorine and bromine.^[^ ^100^ ^]^ However, the common defects for the iodine, the ultra‐small distance between dots and the cause integrated dimer, are depicted by the white circle in Figure 7b.^[^ ^26^ ^]^ Overall, halides show potential for PbS‐QDs ligand exchange, especially the iodine is the lowest defective, but the dots fusion is detrimental to the charge transfer.

To treat the dimer, lead mono‐ or multihalide emerged investigation because it assists in surface homogenization and ceases the hydroxyl ligands (Pb–OH) remained on the surface. The PbI_2_ ligand and iodic acid replace the OA group and reduce the hydroxyl ligands, resulting in free water and traps. Afterward, by utilizing a 0.75:1 I/Pb ratio, this passivation strategy allows thicker absorber film to achieve higher mobility and deliver a PCE of 10.78%. The decrease in hydroxyl ligands facilitates the increase in the absorber thickness by 80 nm.^[^ ^102^ ^]^ Therefore, lead bromide is efficient in removing hydroxyl ligands from the surface, accomplishing a 60% diminution in trap density, a 25% increase in carrier diffusion length, and thus a PCE improvement from 10.9% to 12.5%.^[^ ^93^ ^]^ Additionally, the lowest trap states of 1.5 × 10^15 ^cm^−3^ V^−1^ are a result of preventing the dots fusion, as shown in Figure 7c.^[^ ^93^ ^]^ The bromide holds higher trap density, while iodide causes QDs fusion, so the mixed halides entered the investigation. Therefore, the mixed halides^[^ ^103^ ^]^ are adjusted for LQDs ligand exchange and realized the hybrid ligand strategy by accurately controlling the ratio of bromide to iodide and in situ chloride ion to encounter the (111) and (100) facet passivation. Lead halides are commonly used for IR QDs passivation, such as PbI_2_ and PbCl_2_,^[^ ^103^ ^]^ These anions passivate the interspace gaps and improve the electronic connection between QDs.^[^ ^66^ ^]^ Particularly, PbI_2_ passivation has low efficiency compared to PbCl_2_. Also, PbCl_2_ causes resistance to the charge transfer. Thus, the mixed halogens passivation is more efficient in the LQDs than the single case^[^ ^103^ ^]^

Eventually, the research in ligands has arrived at a plateau state, where the lead halides (PbIBr) stepped up on their peers. Despite this, the nonreactive halides are settled in the interdot space shaping an amorphous phase that disputes the charge transfer. Also, the hybrid halides and Pb*X*_2_ are not entirely suppressing the OH group.^[^ ^62^ ^]^ The Pb*X*_2_ ligand shell is aggregated by the influence of the butylamine solvent used for QDs dispersion and deposition and causes fusion of the non‐passivated QDs.^[^ ^15^ ^]^ In light of the reason above, the lead halide salts require supplementary ligand treatment leading to hybrid ligands.^[^ ^104^ ^]^

#### 3.2.2 Hybrid Ligands

The halides mitigation emerges two challenges in Pb*X*_2_ ligand exchange: the existence of halides in the interdot space and the QDs fusion at the neutral charge (100) facet. These two factors are considered the reason for the mid‐gap trap states as well as the OH group on the surface. So the hybrid ligand exchange^[^ ^15^, ^88^ ^]^ or the postligand passivation^[^ ^8^, ^11^, ^46^ ^]^ is investigated.

Seeking to eliminate the OH group, Gu et al. utilized mercaptopropionic acid (MPA), which strongly interacted with the BA solvent due to the carboxyl and thiol groups.^[^ ^72^ ^]^ This suggests that the incorporation of MPA inhibits mid‐gap states by defending surface oxidation.^[^ ^105^, ^106^ ^]^ In contrast, the zinc iodide and 3‐mercatopropyonic ligands are combined to overcome the *V*_oc_ deficit.^[^ ^55^, ^105^ ^]^ For the same purpose, the ammonium acetate (AA) additive passivates the dangled bongs during lead halide ligand exchange. Recapitulation the benefits of Pb*X*_2_/AA exchange in the smaller interdot distance and obtains narrower distribution. Also, this achieves a flatter energy landscape, reducing both the band tail and Urbach energy.^[^ ^69^ ^]^ As increasing the amount of AA yields QDs fusion, sodium acetate (NaAC) additives with the traditional Pb*X*_2_ introduced and caused improvement in all the facets passivation, particularly the (001) facets are passivated by Na^+^. In contrast, the conventional Pb*X*_2_ passivates the (111) parts.^[^ ^15^ ^]^

The band alignment and lattice matching between PbS‐QDs and CsPb*X*_3_ give the latter a unique weight for bridging the dots and super coupling. However, Pb*X*_2_ is efficient for the polar (111) facet, the dominated (200) facet in the LQDs cannot be passivated due to its neutral charge. CsPb*X*_3_ intersects to bridge the nonpolar facet assisting the Pb*X*_2_ for the (111) facet.^[^ ^82^ ^]^

For dimer prevention, Mandal et al. presented the hybrid of 3‐chloro‐1‐propanethiol (CPT) and PbI^3−^ and passivating the neutral charge (200) facets at which the QDs are fused. As the thiol molecule detains the formation of a halide matrix at the surface, it efficiently prevents dimer and polydisperse QDs.^[^ ^26^ ^]^ In contrast, a simple approach to S^2−^ sites and bromide hybrid passivation on the lead‐rich surface creates S‐crosslinking PbS‐QDs decreasing the roughness of the QDs surface. Bromide and sulfur hybrid‐capping QDs exhibited remarkably enhanced carrier mobility.^[^ ^107^ ^]^ Recently, the thiol and thiocyanate have been introduced as halide‐free ligands to obtain QD film with hole‐high mobility over 0.1 cm^2^ V^−1^ s^−1^. PbS film with improved mobility complement with the other based on lead halide to generate p‐n junction of bulk homojunction PbS‐QDs solar cell achieving a PCE of 10.7%.^[^ ^59^ ^]^ The hybrid passivation with halides can suppress the mid‐gap states by treating the neutral charge (100) facets, suppressing the OH group from the surface.

To wrap up, the ligand exchange is an essential stage in QDs solar cells fabrication process. Attribute to this stage several sources for mid‐gap states such as the QDs fusion, interdot spacing, polydispersity, and nonpassivated facets should be finely treated. Relied on ligand exchange, these defects are passivated, as summarized in Table 1. Furthermore, the postdeposition treatments are discussed in the coming section, including the device's fabrication.

### 3.3 Advancement in the Device Structure

**Figure 8:**
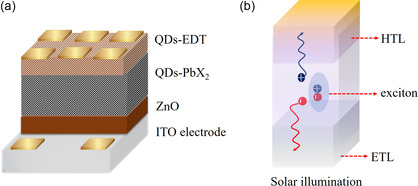
a) Schematic diagram showing the traditional structure for the LQDs solar cell. b) Schematic diagram illustrating the charge‐transfer mechanism through the PbS‐QDs device depicting the interfaces within the structure, and the lines at the interfaces depict the trap states.

Architecture engineering also cooperates in the battle against defects. **Table** **2** summarizes the modified structures over the past 5 years focusing on these performances higher than 10%. The control, ITO/ZnO/PbS CQDs/PbS‐EDT/Au (**Figure** **8**a), is a structure commonly has a zinc oxide layer as ETL that allows electrons and blocks holes to move to the back contact indium tin oxide (ITO). In parallel, the holes extracted through the HTL also block the electron movement to the Au upper contact. In Table 2, the most efficient structure till now has separate passivation strategies for defects at the QDs surfaces and the whole device interfaces.^[^ ^8^ ^]^ The surface and interface defects are the reason for the nonirradiative recombination and the weak charge transfer. Figure 8b explains the optoelectronic processes in the PbS‐QDs device structure. The higher electron mobility than hole’ and trap states at the interfaces are the main challenge that architecture engineering should solve.^[^ ^8^, ^108^, ^109^ ^]^ The advancement in the device structures and the target to cross the efficiency S–Q boundary, are highlighted in this section.

**Table 2:** The common architectures for PbS‐QDs solar cells reported in the recent 5 years, along with their PV parameters

	Architecturea)	*V*_OC_ [V]	*J*_sc_ [mA cm^−2^]	FF [%]	PCE [%]	Year	References
1	ITO/ZnO/PbS‐PbI_2_/PbS‐EDT/Au	0.62	25	70	11	2022	[89]
2	FTO/ZnO/PMMA: PCBM/PbS‐APb*X*_3_/PbS‐EDT/PMMA‐GO/Au	0.66	31.5	74	15.4	2022	[8]
3	ITO/ZnO/BHJ PbS/PbS‐EDT/Au	0.6	32.4	55	10.7	2022	[59]
4	ITO/ZnO/PbS/CsPbI_3_QDs/PTB7‐th/MoO_3_/Ag	0.65	28.6	66	12.6	2022	[115]
5	ITO/ZnO/PbS‐PbIBr/PbS‐EDT/Au	0.49	34.48	62	10.47	2022	[12]
6	FTO/ZnO/ZnO NW/I‐ligand PbS QD/Au	0.53	29.8	60	9.56	2021	[116]
7	ITO/ZnO/CPT−PbS‐QDs/PbS‐NH4SCN/Au	0.6	30.2	58	10.5	2021	[129]
8	ITO/ZnO/PbS‐QDs/PbS‐EDT/MoO_3_/Au/Ag	0.6	30.3	52	9.6	2020	[97]
9	ITO/ZnO/FABr‐PbS/PbS‐EDT /Au	0.65	30	71	13.8	2020	[11]
10	ITO/ZnO/BHJ CQDs PbS/PbS‐EDT/Au	0.65	30.2	68	13.3	2020	[42]
11	ITO/ZnO/CSMAFA‐PbS/PbS‐EDT/Au	0.59	28.9	66	11.3	2020	[9]
12	ITO/ZnO/CQDs PbS/PBDTTT‐E‐T/Au	0.43	5.6	56	6.83b)	2019	[14]
13	ITO/ZnO/CQDs PbS‐TBAI/PbS‐EDT/Au	0.6	25.69	70	10.82	2018	[10]
14	ITO/ZnO/PDMII‐PbS‐PDMII/HAL/Au	0.68	24.11	67	10.99	2017	[128]

a) Tetrabutylammonium iodide (TBAI); poly(methyl methacrylate) (PMMA); [6,6]‐phenyl‐C61‐butyric acid methyl ester (PCBM); poly([2,6′‐4,8‐di(5‐ethyl hexyl thienyl)benzo[1,2‐b;3,3‐b] dithiophene] {3‐fluoro‐2[(2‐ethylhexyl)carbonyl] thieno[3,4‐b] thiophenediyl) (PTB7‐Th); 1‐propyl‐2,3‐dimethyl imidazolium iodide (PDMII); methyl mercapto propionate (MPE); PBDTTT‐E‐T, a copolymer of benzothiophene (BDT) and thienothiophene;

b) This is an inferred PCE.

The inverted structure, ITO/HTL/absorber/ETL/metal electrode, has achieved efficiency limited to 9.7% since January 2018.^[^ ^110^ ^]^ In this structure, the research scope is to select the convenient hole buffer layer. In particular, the NiO, V_2_O_5_, and PEDOT:PSS layers are HTLs, while the PCBM is usually used as ETL in the inverted structures.^[^ ^111^ ^]^ However, the ZnO film is applied as ETLs in the most efficient PbS‐QDs inverted solar cell that had ever been fabricated, which was mentioned before.^[^ ^110^ ^]^ Also, the solution‐processable cadmium sulfide was investigated as ETL in the FTO/CdS/PbS‐QDs/MoO_3_/Ag structure, achieving a PCE of 5.22%.^[^ ^112^ ^]^ Notably, the focus on the inverted PbS‐QDs solar cells has diminished recently. Last year, there was no reported research on the inverted structure PbS‐QDs solar cells, so our review concentrates on the standard design of normal structure.

#### 3.3.1 Absorber Deposition and Post‐Treatment

Till now, the deposition of QDs absorber requires a controlled inert atmosphere. It happened in a nitrogen‐filled glovebox to stabilize the core/shell structure and block the influence of humidity. Then film annealing evaporates the organic molecules. In any case, postheating removes the long organic chain from the QDs surface and improves the performance. Also, the postheating assists in raising the conduction band minimum (CBM) to bend with the ZnO band. By restraining the temperature, the LQDs become closer, increasing the *V*_oc_ and improving the charge transfer,^[^ ^113^ ^]^ increasing the initial PCE by 38%. Alternatively, the remained organic ligands and defects on the PbS‐QDs device are removed by applying oxygen plasma to the surface of PbS.^[^ ^114^ ^]^ Similarly, the deposition of a thin polymer film improves the carrier transfer and performance by more than 50% compared to the untreated device.^[^ ^71^ ^]^ The absorber deposition and treatment are critical for trap state prevention. Chemically, merging the film in supporting solution or deposition of interfacial layer shows potential in trap passivation as follows.

**Figure 9:**
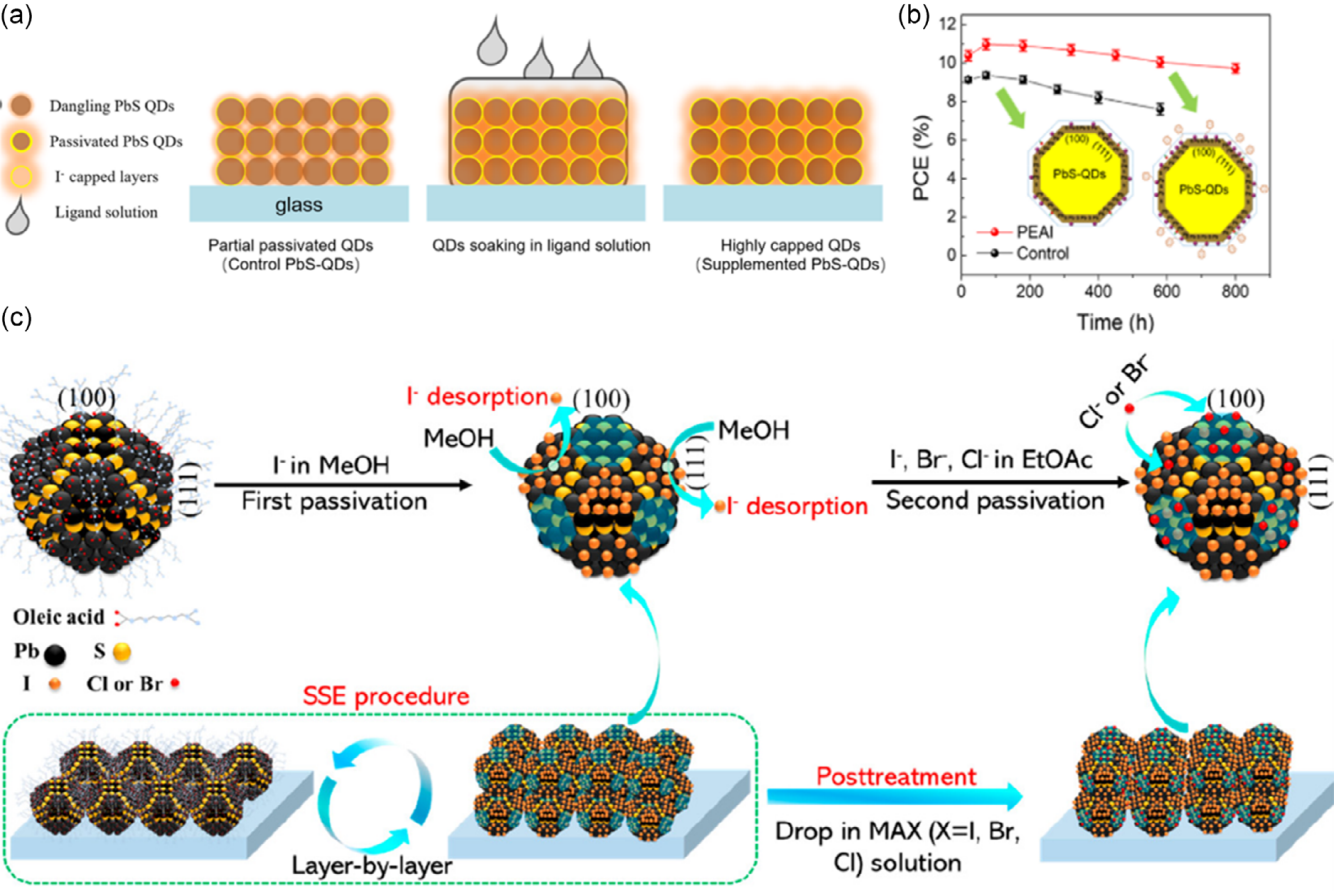
a) Illustration of the different solution methods for organic ammonium iodides. b) The effect of the ambient atmosphere on the performance of the fabricated devices. a,b) Reproduced with permission.^[^ ^76^ ^]^ Copyright 2020, American Chemical Society. c) The absorber, PbS‐QDs treated with iodide ion ligand, deposition and postpassivated by SSE. Reproduced with permission.^[^ ^46^ ^]^ Copyright 2020, American Chemical Society.

After fabricating the QDs film with PbI^3−^ ligand exchange, it was merged in an extra PEAI solution, as shown in **Figure** **9**a. This second stage ligand forms a homogenous film matching the bandgap with the whole structures by PEAI infiltrating the dangled bonds. Thus, the trap density decreased from 7.12 × 10^15^ cm^−3^ to 2.83 × 10^15^ cm^−3^, and the carrier lifetime increased threefolds. Subsequently, the PCE increased by about 20% and worked 200 hrs more than the control device, as shown in Figure 9b.^[^ ^76^ ^]^

Notwithstanding, the methylammonium halides further treated the absorber after the iodide solid‐state exchange (SSE) that passivates the dangled bonds without changing the VBM or CBM. This solid‐state passivation mainly reduces the trap density from 1.7 × 10^17^ to 0.94 × 10^17^ cm^−3^ for electrons and from 3.14 × 10^17^ to 1.86 × 10^17^ cm^−3^ for holes as well. Consequently, the carrier mobility, lifetime, and diffusion length were increased threefold, longer than one ns and 70%, respectively.^[^ ^46^ ^]^ Figure 9c schematically illustrates the two steps of SSE in which the I^−^ ions dissolved in MeOH first passivate the film. Then MACl in ethyl acetate (EtOAc) is applied for additional treatment. The three methylammonium halides, Cl, Br, and I, were also studied. However, the MACl achieves the optimum performance.^[^ ^46^ ^]^

#### 3.3.2 Perovskite Bridge

The perovskite materials in the PbS‐QDs passivations are multifunctional. In some reports, it is used as a supplementary ligand for the (100) facets passivation.^[^ ^82^ ^]^ In others, it is used as an additive for the QDs absorber,^[^ ^8^ ^]^ post‐treatment for the absorber,^[^ ^11^, ^76^ ^]^ and interfacial layer between the QDs and the hole transparent layer (HTL).^[^ ^115^ ^]^ To bridge the (100) facet and resist the dimer initiation, a thin perovskite (PSK) layer coated over QDs films^[^ ^66^ ^]^ such as CsPbI_3_,^[^ ^115^, ^116^ ^]^ FAPb*X*_3_,^[^ ^8^, ^11^ ^]^ triple‐cation perovskite,^[^ ^9^ ^]^ or CH_3_NH_3_PbI_3_ in 2,6‐difluoro pyridine.^[^ ^117^ ^]^ This perovskite bridged QD film with an optimized thickness of about 440 nm is a promising solution for the weak charge transfer between the QDs. As it increases the coupling between QDs and diminishes the distance of tunneling.^[^ ^11^ ^]^ Furthermore, it induces the nucleation of QDs along the (111) direction, making uniform lattice spacing throughout the composite. Thanks to PSK layer, PbS‐QDs achieved the satisfied PCE.^[^ ^8^, ^11^ ^]^

**Figure 10:**
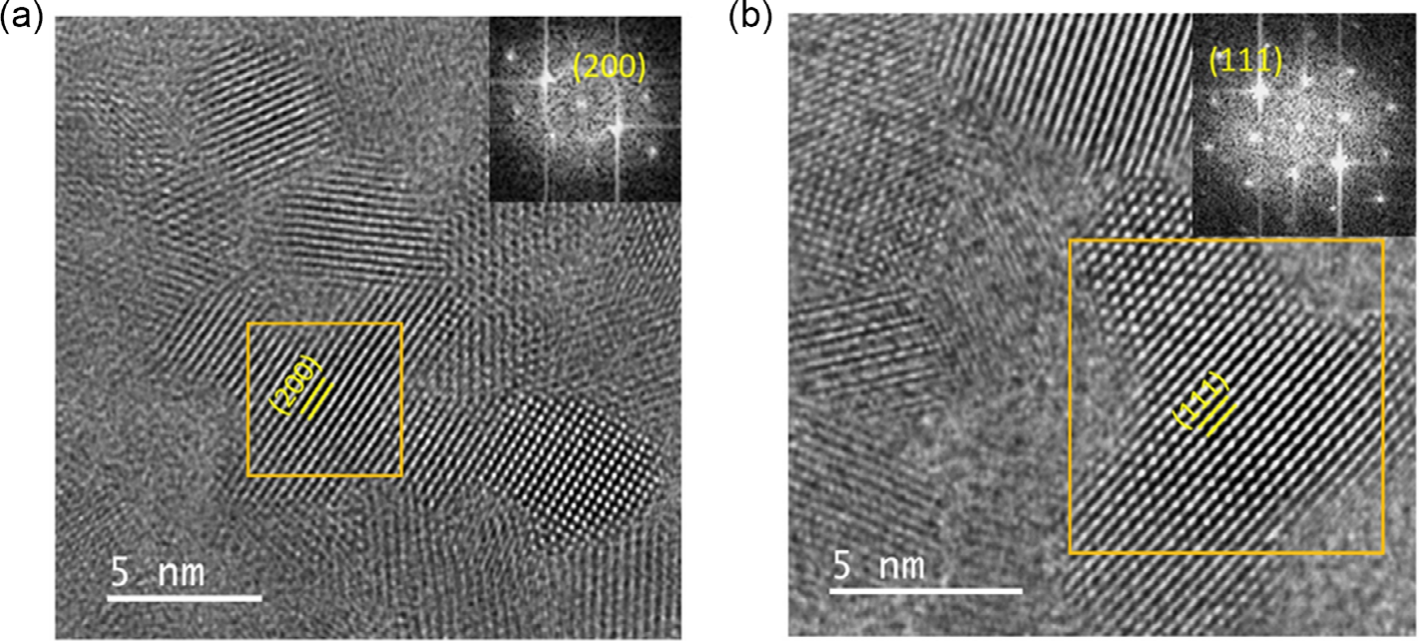
a,b) HRTEM images of an epitaxial CQD cluster connected along the (200) facet (a) and the (111) facet (b), the inset of each image displays the selected‐area electron diffraction (SAED) image. a,b) Reproduced with permission.^[^ ^11^ ^]^ Copyright 2020, Elsevier.

Postcoating of the FABr layer epitaxially develops the PSK monolayer, which connects the QDs, so solar cells obtained a stable PCE of 13.8% due to more extended diffusion length.^[^ ^11^ ^]^ The function of the PVS layer in coupling (200) and (111) facets is, respectively, shown in **Figure** **10**a,b. Also, the interdot space is decreased. For the same purpose, the MAPbI_3_ thin layer is postdeposited, increasing the absorption of PbS‐QDs in the band of 330–1,400 nm and suppressing damage on their surface.^[^ ^118^ ^]^

#### 3.3.3 Passivation of ETL and Its Interface

**Figure 11:**
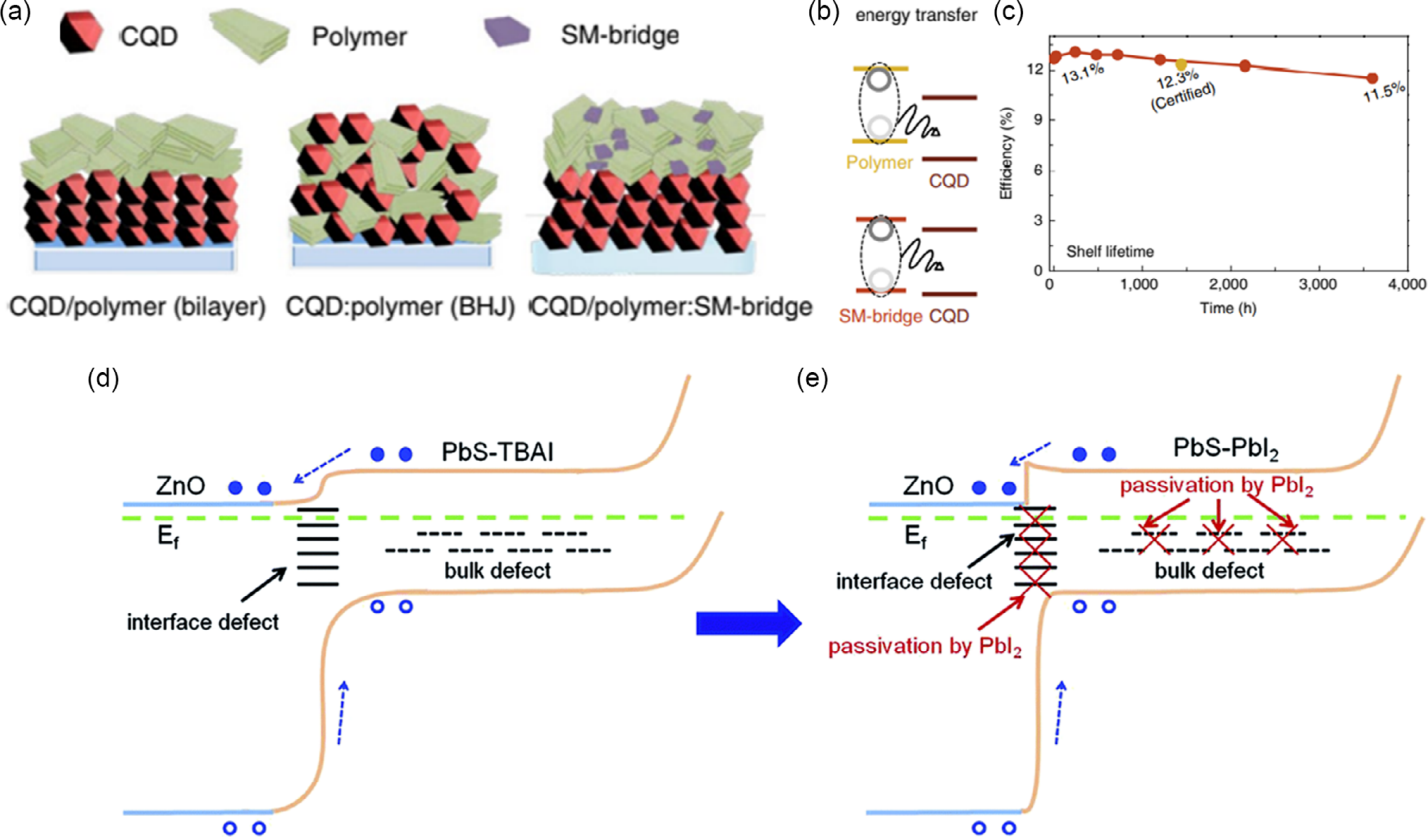
a) Schematic illustration of the CQDs’ surface modification composed of a polymer layer and small molecule (SM)‐bridge deposition. b) The benefits of SM and polymer combination on charge transfer from their levels to the conduction and valance band of the PbS‐QDs. c) The PCE reduction of the fabricated devices kept under an ambient atmosphere. a–c) Reproduced with permission.^[^ ^119^ ^]^ Copyright 2019, Springer Nature. d) Energy–band diagram for the control PbS‐QDs device and e) after deposition of PbI_2_ passivation layer. d,e) Reproduced under the terms of the CC‐BY Creative Commons Attribution 3.0 Unported license (https://creativecommons.org/licenses/by/3.0).^[^ ^64^ ^]^ Copyright 2017, Royal Society of Chemistry.

Efficient charge extraction and transfer is an advanced priority in PV. Hence, the progress in ETL deposition is a concern, as well as the interfacial defects between the QDs layer and the ETL.^[^ ^105^ ^]^ To enrich the charge transfer, a bridge of small molecules is fabricated in the interspaces of a polymer with unique properties such as excitons‐rich, efficiently transferring energy, and encouraging exciton removal at the interfaces. The schematic diagram in **Figure** **11**a explains the treatment process, in which the polymer layer with the small organic molecule plays the role of the bridge for the carrier diffusion in Figure 11b. This device renders PCE of 13.1%, and the cell remained stable at more than 80% of its efficiency despite continuous operation for more than 150 hrs, as shown in Figure 11c.^[^ ^119^ ^]^

The IS and the β‐recombination model evidenced the dense recombination in PbS‐QDs as the principal cause for the limited *V*_oc_.^[^ ^120^, ^121^ ^]^ Most commonly, the self‐assembly monolayer between the ETL and the absorber narrows the *V*_oc_ deficit. Therefore, dense efforts are spent elongating the electron diffusion length by depositing a thin interfacial layer between the PbS‐QDs and the window layer.^[^ ^64^, ^108^, ^122^ ^]^ This decreases the heterojunction interface and PbS‐QDs surface traps.^[^ ^64^ ^]^ Figure 11d depicts the bandgap of the control device compared to the other passivated lead iodide layer shown in Figure 11e. Also, the thickness of the ZnO layer (the most common ETL in PbS‐QDs solar cells) and doping were optimized at an intermediate thickness of ≈90 nm and had lower resistivity.^[^ ^123^ ^]^

#### 3.3.4 Passivation of the Interface with the HTL

Researchers also consider the interfacial defects between PbS‐QDs and HTL. For instance, a layer of In_2_S_3_ over the PbS film raised its optoelectronic and morphological properties.^[^ ^124^ ^]^ Furthermore, the optimized CdS thickness of 400–600 nm showed increased EQE, slow voltage decay, and improved lifetime. This proves that CdS interfacial layer removes the surface's traps and suppresses recombination.^[^ ^125^ ^]^ The polymers are commonly applied in the PbS‐QDs solar cells as HTLs, but it initiates recombination centers at the interface. Therefore, the deposition of a perovskite layer before its deposition increases the device PCE from 10.5 to 12.32%.^[^ ^115^ ^]^ The recommended interfacial layer's energy band is situated between the PbS‐QDs and HTL to cause well band alignment and dipole charge distribution. Thus, the collection of carriers is raised and substantially transferred.^[^ ^115^ ^]^ In contrast, the cation exchange was applied on the perovskite passivation layer Cs_0.05_(MA_0.17_FA_0.83_)_0.95_Pb(I_0.9_Br_0.1_)_3_ to stabilize the device and maintain about 96% of the initial device performance after storage for about 1200 h. Furthermore, the optimum thickness for the perovskite film obtaining the highest PV parameters is around 350 nm.^[^ ^9^ ^]^ Moreover, considering the band bending and the defects passivation of the QDs layer, the perovskite film motivates the growth of the desired (100) plane of the PbS layer.^[^ ^75^ ^]^

## 4 The Challenges for Industrial Production

Commercial production is still banned by obstacles such as recycling the dense solvents used in synthesis, ligand exchange processes, and quantum dot cleaning. These solvents mainly harm the environment and humans. Further, the resultant QDs mass is limited even by the complicated process, but the industry requires massive production. This large‐scale production encourages developing novel approaches with simple steps to achieve dense and efficient QDs. In contrast, the experimental design for the fabrication steps is controlled under certain conditions to protect the environment and experiment from each other. In particular, preventing harmful materials leaked into the atmosphere is as important as managing the experiment under certain atmospheric conditions. The structure preparation approaches are restricted to the tiny scale device. Scalable fabrication requires different techniques, such as roll‐to‐roll and blade deposition, etc.

Finally, the PbS material harms the environment, which must be solved before commercial production. The solution could be implemented by applying conductive and transparent polymer materials that insulate the toxicity from contacting the environment. This will improve the stability of the device as well. Alternatively, scientists need to focus on discovering green QDs to replace those that contain heavy metals. The stability of the PbS‐QDs device is still shallow compared with the industry requirements.

## 5 Conclusion

IR absorbers can optimize the use of solar‐radiated energy. Besides, they are good candidates as subcell in crystalline silicon or perovskite within the tandem structure. PbS‐QDs solar cell is not the only IR absorber, but it is cheaper and could be easily prepared comparable to silicon, Ga‐arsenic, and other IR absorbers. Even though its efficiency is limited by 15% because of the normal trap states acting as recombination center. Consequently, it causes *V*_oc_ deficit and retard the photovoltaics’ performance improvement. Many defect treatment strategies were investigated in various studies. This review summarized these strategies, highlighting the optimal performance, straightforward preparation techniques, and low defect concentration. First, the focus on CQDs synthesis methods has obtained the highest PCE value of 14% by developing a self‐programming synthesis method. It is recommended to replace the long organic molecule (OA) with other inorganic atoms to filtrate the vacancies on the QDs surfaces on the branch of ligand exchange. Halides and mixed halides strategies were introduced to give high performance. Controlling the CQD size is the key to tuning the bandgap and, subsequently, the absorption. It could be achieved with the assistance of temperature factors influencing the kinetic concerning thermodynamic growth.

Furthermore, the poor passivated (100) facet that accompanied the desired size for IR solar cells was treated by the introduction of cation exchange from ZnS nanorods and supporting ligands along with the conventional one. Conversely, it is recommended to cure the ETL and HTL similarly to the absorber CQDs. Thus, the reported sputtering method to deposit the ZnO is recommended to improve interface quality.

For the future of PbS CQDs, it has several channels to compete with traditional and expensive solar cell devices. Prospectively, CQD efficiency has to meet the theoretical bound by applying the mentioned strategies individually or combined. Similarly, control of electrons and hole motion on the device should take comprehensive efforts in future work. Hence, the infrared based on the PbS‐CQDs would have sufficient efficiency to be an economically industrial product. The optimization of the CQDs solution and films will be continued in the future, along with the discovery of novel convenient ligands that will eventually reduce the trap density to a sufficiently low level for crossing the PCE limit. The decrease in the defect density by one order of magnitude is reported to increase the *V*_oc_ ≈ 75 mV and more than 15% PCE if the FF and *J*_sc_ are constant.^[^ ^25^ ^]^ Besides, LQDs utilization as smart windows for buildings in warm cities shows high potential. IR radiation's influence on global warming is well known. It provides power for the buildings and reduces the need to operate the air conditioners.

The production cost for tandem PV should be decreased, and the structure based on PbS‐QDs needs optimization in future work. For instance, choosing the transparent and conductive interconnection layer is significant to act as photonic and plasmonic material. The transmittance of light through all the tandem layers and elongating diffusion of the generated carriers produce efficient tandem devices. Keeping attention to preventing the recombination centers in the PbS‐QDs is our recommendation to construct competitive tandem devices based on PbS‐QDs.

## Conflict of Interest

The authors declare no conflict of interest.
